# Supplementary material for: Chronic Bronchitis Affects Outcomes in Smokers without Chronic Obstructive Pulmonary Disease (COPD)
Source: J Clin Med. 2022 Aug 20;11(16):4886. doi: 10.3390/jcm11164886 (PMC9410001; doi:10.3390/jcm11164886)
Supplement: Supplementary file 1 [file jcm-11-04886-s001.zip › jcm-1819071-supplementary.pdf]

## Supplementary Data

# Chronic bronchitis affects outcomes in smokers without chronic obstructive pulmonary disease (COPD)

Alvise Casara, Graziella Turato, Marta Marin-Oto, Umberto Semenzato, Davide Biondini, Mariaenrica Tinè, Nicol Bernardinello, Elisabetta Cocconcelli, Pablo Cubero, Elisabetta Balestro, Paolo Spagnolo, Josè Maria Marin, Manuel G Cosio, Marina Saetta, Erica Bazzan

## Methods

### *Patient population*

Participants were recruited consecutively among smokers (>10 pack/years) who first attended the Pulmonary Clinic at the Hospital Universitario Miguel Servet (Zaragoza, Spain) between October 2010 and April 2014. The objective of constituting this cohort was to determine health related outcomes in smokers with and without COPD, free of major comorbidities (defined as chronic conditions that needed regular therapy: e.g, diabetes, hypertension, dyslipimia) at recruitment. Eligible individuals were >40 years old smokers who came to the clinic requesting to be included in our smoking cessation program or referred by other doctors to assess their respiratory health. Patients who agreed to be included in the cohort signed an informed consent before any procedure was performed.

Of the 2453 smokers who came to the outpatient respiratory clinic during the recruiting period, 1130 subjects did not meet the inclusion criteria (>40 years, <10 pack/years), and 802 were excluded because of concomitant comorbidities. Among the remaining 521, 10 subjects were lost during follow-up, and 511 participants were included in the analysis; 302 with COPD and 209 without COPD (noCOPD) (Fig.E1).

All subjects underwent functional and clinical examination including pulmonary function tests, modified Medical Research Council (mMRC) dyspnea and COPD Assessment Test (CAT) scores evaluation. Exacerbations were collected and defined as acute worsening of respiratory symptoms that required antibiotics and/or oral corticosteroids (moderate) by medical prescription, or hospitalization/visit at the emergency room (severe) [E1]. The annual exacerbation rate of each patient was calculated by dividing the number of exacerbations by the number of days they participated in the study, and multiplying by 365.

**Figure S1: CONSORT diagram. Flow chart of the study design**

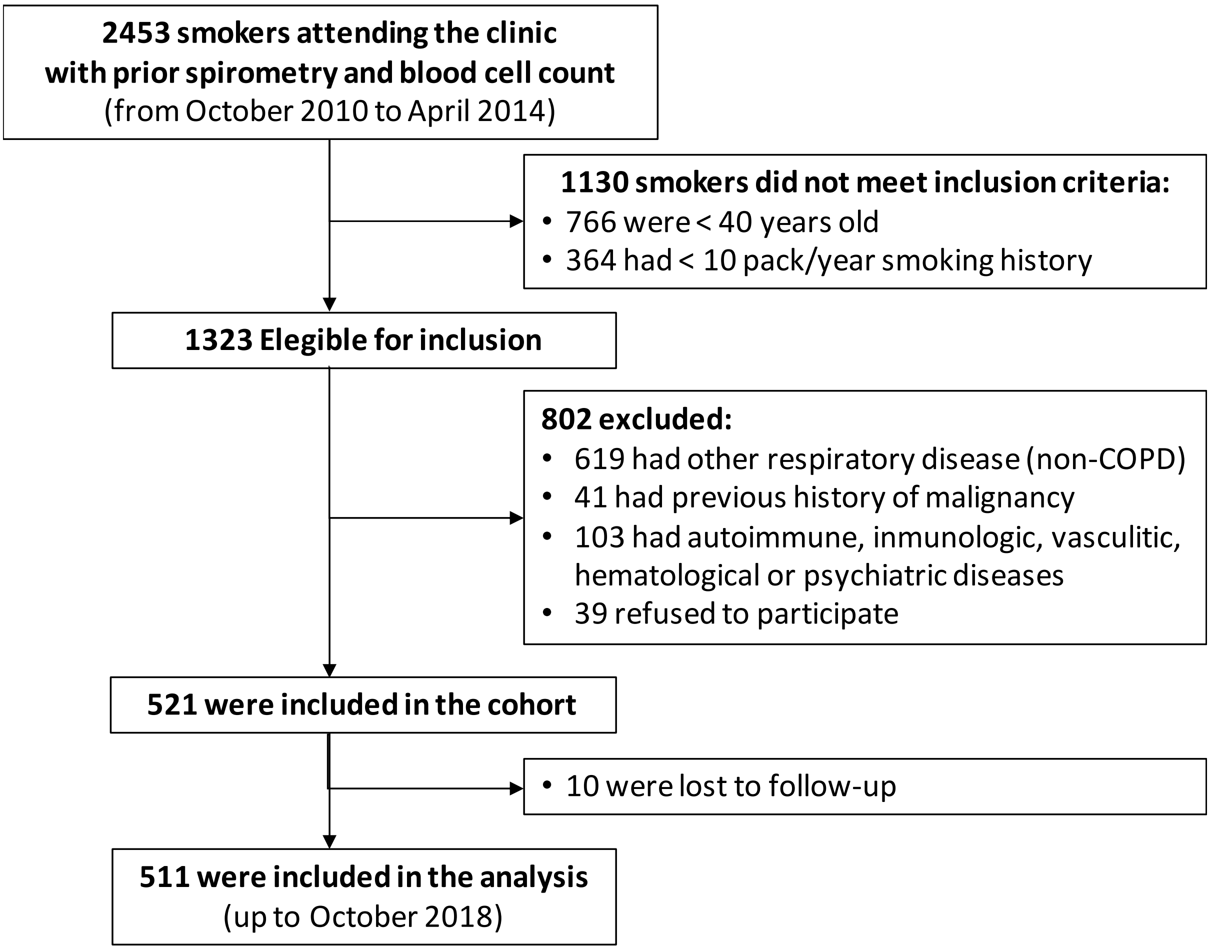

**Figure S2. Frequency of exacerbations in follow-up period in noCOPD (A) and COPD (B).**

A

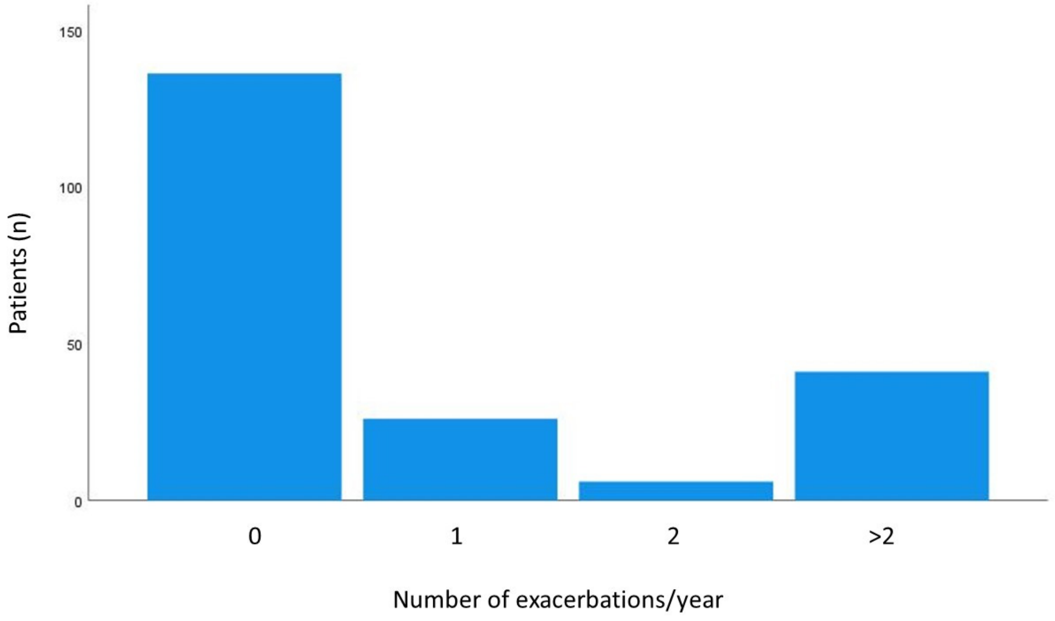


B

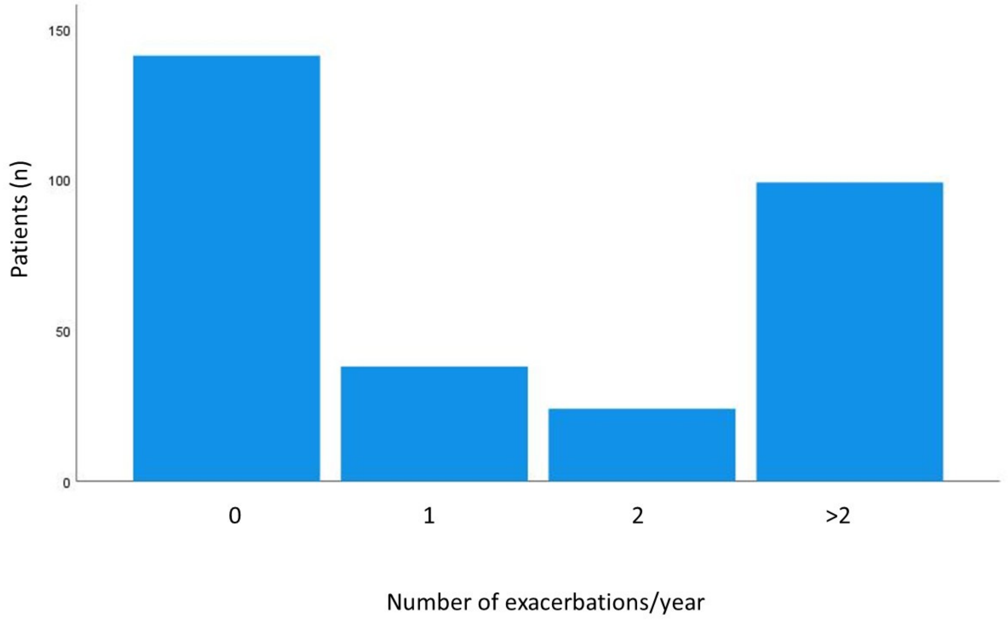

**Table S1. Therapy in the COPD with and without CB**

| Therapy | COPD (n=302) | COPD w/o CB (n=165) | COPD with CB (n=137) | P |
|---|---|---|---|---|
| **ICS** | 179/302 (80%) | 96/165 (58%) | 83/137 (60%) | ns |
| **LAMA** | 35/302 (11%) | 20/165 (12%) | 15/137 (11%) | ns |
| **LABA/LAMA** | 10/302 (3%9) | 6/165 (4%) | 4/137 (3%) | ns |
| **LAMA/ICS** | 52/302 (17%) | 31/165 (19%) | 21/137 (15%) | ns |
| **TripleTherapy** | 120/302 (40%) | 59/165 (36%) | 61/137 (44%) | ns |

*Data are presented as number (%). p value refers to Mann-Whitney test or χ2 test*

## SUPPLEMENTARY REFERENCES

E1. Global Strategy for the Diagnosis, Management and Prevention of Chronic Obstructive Pulmonary Disease, Global Initiative for Chronic Obstructive Lung Disease (GOLD) 2021. goldcopd.org
